# DEVELOPING A PRACTICAL, SCALABLE QI INITIATIVE FOR FQHCS IN WASHINGTON STATE: THE COGNITION IN PRIMARY CARE PROGRAM

**DOI:** 10.1093/geroni/igae098.0424

**Published:** 2024-12-31

**Authors:** Barak Gaster

**Affiliations:** University of Washington, Seattle, Washington, United States

## Abstract

In November 2020, the University of Washington (UW) was awared funding from the Centers for Disease Control (CDC) to adapt the Gerontological Society of America’s K.A.E.R. Toolkit for implementation in a large health system. Using focus group interviews, we developed a quality improvement initiative, Cognition in Primary Care (CPC), consisting of concise continuing education (CE) for primary care providers (PCPs) paired with tools to put that training into practice. The CE consists of Webinars which introduce the tools, which take the form of electronic health record (EHR) checklists and resource folders to facilitate performing cognitive evaluations in primary care. Between 2021 and 2023, the CPC program was implemented in 14 community-based primary care clinics at UW. Measures to assess outcomes showed positive effects including uptake of the EHR tools by PCPs, increased cognitive-tests entered into the EHR, and increased number of patients identified with cognitive impairment. During the symposium, attendees will learn the components of the CPC approach for quality improvement, as well as the methodology used to implement it and assess its effects. Finally, we will present an overview for how the CPC model is being packaged for dissemination to health systems across Washington State, with a focus on federally qualified health centers, supported by new CDC funding in the form of a grant through the BOLD Public Health Initiative.

